# Supplementary material for: Health care managers’ perspectives on workforce licensing practice in Ethiopia: A qualitative study
Source: PLoS One. 2024 Apr 18;19(4):e0302122. doi: 10.1371/journal.pone.0302122 (PMC11025822; doi:10.1371/journal.pone.0302122)
Supplement: S1 File — (DOCX) [file pone.0302122.s001.docx]

COVER LETTER

**Plos One**

**Date: 04/04/2024**

**Title: Health care managers’ perspectives on workforce licensing practice in Ethiopia: A qualitative study”, PONE-D-22-33343R3, DOI: 10.1371/journal.pone.0302122**

Dear Plos one team, we are so glad to inform you that we submitted our revised manuscript as per the plos one journal guideline by addressing the given comments. We really acknowledge you for giving us reviews and edits of our manuscript for the betterment of our work. The corresponding author of this manuscript is **Eshetu Cherinet Teka (MPH)** and any communication will be held with him. We would also like to include **Endalkachew Tsedal Alemneh (MPH-N) and Meron Yakob Gebreyes (DDM, MPH**) as a corresponding author based on your multi corresponding author policy.

We tried to address all the comments given. We attached the manuscript (untracked), manuscript (tracked), letter(line by line response to comments). Kindly, we are ready to correct any of your comments at any time.

Thank you!

Eshetu Cherinet (MPH)
