# Supplementary material for: Health care managers’ perspectives on workforce licensing practice in Ethiopia: A qualitative study
Source: PLoS One. 2024 Apr 18;19(4):e0302122. doi: 10.1371/journal.pone.0302122 (PMC11025822; doi:10.1371/journal.pone.0302122)
Supplement: S2 File — (DOCX) [file pone.0302122.s002.docx]

Top of Form

**View Letter**

| **Date:** | Apr 01 2024 03:11AM |
| --- | --- |
| **To:** | "Eshetu Cherinet Teka" eshecher2005@gmail.com |
| **From:** | "PLOS ONE" plosone@plos.org |
| **Subject:** | Required Modifications for PONE-D-22-33343R3 |
| **Attachment(s):** | [PONE-D-22-33343R3_FTC.docx](https://www.editorialmanager.com/pone/download.aspx?guid=%7bc78af264-e062-4a48-b23e-0f6e0182e21f%7d&scheme=13&id=528351) |

PONE-D-22-33343R3
Health care managers’ perspectives on workforce licensing practice in Ethiopia: A qualitative study

Dear Dr. Teka:

Thank you for submitting your work to PLOS ONE. Your manuscript will be formally accepted and enter production after you complete the requests below. Please note that you will not be able to make changes to your manuscript once it enters the production process. PLOS ONE does NOT provide author proofs. Any changes other than those requested in this email will need to be reviewed by the Academic Editor and reviewers; this will delay the formal acceptance of your manuscript.

To access your manuscript and complete these changes, please follow this link: ********.

Please note that this link should not be shared with other people to ensure that any action taken on this submission is done by you, the Corresponding Author.

You will find the submission in "Current Task Assignments." From there you must download your submission files via the "Assignment Files" link. You can also download the manuscript file attached to this email; the document has been formatted to track all changes, and we will only accept this version with locked tracked changes returned to us.

To opt in or out of publishing your peer review history, please answer the Peer Review History question within this task. Please note we are unable to send your article to production without this.

Once you have made all the required changes, click "Submit Task" to upload the corrected files.

Your task is due Apr 04 2024 11:59PM.

Please contact plosone@plos.org with any questions or concerns. For billing related questions, please contact our Author Billing department directly at authorbilling@plos.org. For questions regarding your press release or the press process, please contact onepress@plos.org

With kind regards,
PLOS ONE staff

JOURNAL REQUIREMENTS:

1. Please ensure that the author list and affiliations are correct on the title page of your manuscript, and that your author contributions, competing interests, and financial disclosure are correct as listed below. All of these sections will be indexed in PubMed and published by PLOS ONE as you have written them. Please email plosone@plos.org if any changes to this content need to be made.

Eshetu Cherinet Teka:
Formal analysis
Investigation
Methodology
Writing – original draft
Writing – review & editing

Meron Yakob Gebreyes:
Formal analysis
Investigation
Methodology
Writing – original draft

Response: Meron Yakob Gebreyes is the corresponding author as per your multiple corresponding author policy

Endalkachew Tsedal Alemneh:
Formal analysis
Investigation
Methodology
Writing – original draft
Writing – review & editing

Response: Endalkachew Tsedal Alemneh is the corresponding author as per your multiple corresponding author policy

Biruk Hailu Tesfaye:
Formal analysis
Investigation
Methodology
Writing – original draft
Writing – review & editing

Firew Ayalew Desta:
Methodology
Writing – original draft
Writing – review & editing

Yohannes Molla Asemu:
Conceptualization
Formal analysis
Methodology
Visualization
Writing – original draft
Writing – review & editing

Ermias Gebreyohannes Wolde:
Investigation
Methodology
Writing – original draft
Writing – review & editing

Wondimu Daniel Ashena:
Investigation
Methodology
Writing – original draft

Samuel Mengistu:
Investigation
Methodology
Writing – original draft

Tewodros Abebaw Melese:
Methodology
Project administration
Supervision
Writing – original draft

Fikadie Dagnew Biset:
Investigation
Project administration
Writing – original draft

Bezawit Worku Degefu:
Investigation
Methodology
Supervision
Writing – original draft
Writing – review & editing

Bethlehem Bizuayehu Kebede:
Formal analysis
Methodology
Writing – original draft
Writing – review & editing

Tangut Dagnew Azeze Azeze:
Investigation
Methodology
Writing – original draft

Response: The above repeated grand father’s name “Azeze” is removed

Wudasie Teshome Shewatatek:
Investigation
Methodology
Writing – original draft

Melese Achamo Seboka:
Investigation
Methodology
Writing – review & editing

Abera Bezabih Gebreegzi:
Investigation
Methodology
Writing – review & editing

Mekonnen Dessie Degebasa:
Formal analysis
Methodology
Writing – original draft

Response: The above name Dessie is corrected by Desie

Tsedale Tafesse Lemu:
Formal analysis
Methodology
Writing – original draft

Yeshiwork Eshetu Abebe:
Investigation
Methodology
Writing – original draft

Matias Azanaw Alayu:
Investigation
Methodology
Writing – original draft

Fatuma Ahmed Ebrahim:
Investigation
Methodology
Writing – original draft

Eden Workneh Sahlemariam:
Investigation
Methodology
Writing – original draft

Genet Kifle woldesemayat:
Investigation
Methodology

Hailemaryam Balcha Admassu:
Investigation
Methodology
Writing – original draft

Bethlehem Shikabaw Chekol:
Investigation
Methodology
Writing – original draft

Please see here for the full list and definition of contributor roles: http://journals.plos.org/plosone/s/authorship#loc-author-contributions

Please ensure that the Competing Interests and Financial Disclosure statements listed below are suitable for publication. These sections will be indexed in PubMed and published by PLOS ONE as you have written them. Please email plosone@plos.org if any changes to these statements need to be made.

Competing Interests:
The authors have declared that no competing interests exist.

Financial Disclosure:
The author(s) received no specific funding for this work.

Response: Thank you! Both Competing Interests and Financial Disclosure statements are suitable for publication.

2. Some of authors name listed in your submission paper is different from the authors name listed in the online submission. Please let us know which names should we use for your submission.

Corrected I number one above. i.e. Response: The above repeated grand father’s name “Azeze” is removed

Response: The repeated grand father’s name “Azeze” is removed and the father’s name “Dessie” is corrected by Desie.

3. Please provide the complete details of the affiliations. Please include each component in order of small to large:

• Department/Division/Section | Institution | City | State (if applicable) | Country

Please refer to our downloadable sample files to make sure that your affiliations and byline are formatted correctly: <http://journals.plos.org/plosone/s/file?id=ba62/PLOSOne_formatting_sample_title_authors_affiliations.pdf>

Response: Thank you! it is done as per the comment in the manuscript and in the system

4. We note that you have included a Funding Statement within the body of your manuscript. Please note that declarative statements should not be included within the manuscript. Please ensure that the statement provided in the submission system is correct, and then remove the statement from your manuscript file.

Response: Funding statement is removed from the manuscript and the statement in the system is correct

5. Please remove "Author Contributions" in your paper.

Response: Thank you! removed as per the comment

6. We note that you have included a Competing Interests Statement within the body of your manuscript. Please note that declarative statements should not be included within the manuscript. Please ensure that the statement provided in the submission system is correct, and then remove the statement from your manuscript file.

Response: Great thanks! Competing Interests Statement is removed from the manuscript as per the comment

7. We note that you have included a Data Statement within the body of your manuscript. Please note that declarative statements should not be included within the manuscript. Please ensure that the statement provided in the submission system is correct, and then remove the statement from your manuscript file.

Response: Thanks! We removed data statement from the body of the manuscript and check in the system is correct

8. In the text, cite the reference number in square brackets (e.g., “We used the techniques developed by our colleagues [19] to analyze the data”).

Response: the reference in brackets () are changed to square brackets []

9. To prevent production delays, we recommend using the Author Formatting Checklist to confirm that your paper meets PLOS ONE's typesetting requirements for References, Tables, and Figures: <http://journals.plos.org/plosone/s/file?id=c819/plos-one-author-formatting-checklist.docx>.

This checklist is a reference tool for you; please do not upload the completed Author Formatting Checklist with your submission files.

Response: Ok! Thank you! It is checked and corrected accordingly.

10. To ensure your figures meet our technical requirements, please run each figure included in your submission files through the PACE tool: https://pacev2.apexcovantage.com/. PACE will assess whether your figures meet our technical requirements and will fix the figure(s) or identify any problem(s) that cannot be automatically fixed. It can also convert figures to TIFF format, resize, and rename figures to meet our naming conventions.

Response: Thank you! We have no figures.

To use PACE, first register as a user. Follow the instructions on the site for assessing and converting your figure files. If you experience any difficulty using this tool or have questions about any of the figures and/or images in your paper, please inform the journal office in your response letter.

CONFIDENTIAL: This email and any attachments are confidential and for the sole use of the individual(s) to whom they are addressed. If you have received this message in error please delete the message and notify plosone@plos.org.

__________________________________________________
In compliance with data protection regulations, you may request that we remove your personal registration details at any time. (Use the following URL: https://www.editorialmanager.com/pone/login.asp?a=r). Please contact the publication office if you have any questions.

Bottom of Form

Explain

Translate

After selecting text, you can set a shortcut key to display the Context Menu.

Got itGo to Settings
