# Supplementary material for: Health care managers’ perspectives on workforce licensing practice in Ethiopia: A qualitative study
Source: PLoS One. 2024 Apr 18;19(4):e0302122. doi: 10.1371/journal.pone.0302122 (PMC11025822; doi:10.1371/journal.pone.0302122)
Supplement: Attachment — Submitted filename: Response to reviewer.docx [file pone.0302122.s003.docx]

PONE-D-[22-33343](tel:22-33343)
Health care managers’ perspectives on workforce licensing practice in Ethiopia: A qualitative study
PLOS ONE

Dear Eshetu Cherinet ,

Thank you for submitting your manuscript to PLOS ONE. After careful consideration, we feel that it has merit but does not fully meet PLOS ONE’s publication criteria as it currently stands. Therefore, we invite you to submit a revised version of the manuscript that addresses the points raised during the review process.

ACADEMIC EDITOR: 
I agree with the reviewers' comments and advise the authors to address all of them or provide justifications 
Indicate the contributions of each author based on the ICMJE | Recommendations | Defining the Role of Authors and Contributors here (<https://www.icmje.org/recommendations/browse/roles-and-responsibilities/defining-the-role-of-authors-and-contributors.html> )

Response: Thank you! Contribution of each author is based on the ICMJE Recommendations.

Qualitative research studies should be reported in accordance to the consolidated criteria for reporting qualitative research (COREQ) checklist or Standards for reporting qualitative research (SRQR) checklist. Further reporting guidelines can be found in the Equator Network's Guidelines for reporting qualitative research. Complete and attach a relevant checklist for the qualitative study.

Response: Done as per COREQ checklist
Seek language editing support to improve the readability and clarity of the paper.

Please submit your revised manuscript by May [25 2023 11](tel:25%202023%2011):59PM. If you will need more time than this to complete your revisions, please reply to this message or contact the journal office at [plosone@plos.org](mailto:plosone@plos.org). When you're ready to submit your revision, log on to <https://www.editorialmanager.com/pone>/ and select the 'Submissions Needing Revision' folder to locate your manuscript file.

Please include the following items when submitting your revised manuscript:

A rebuttal letter that responds to each point raised by the academic editor and reviewer(s). You should upload this letter as a separate file labeled 'Response to Reviewers'.
A marked-up copy of your manuscript that highlights changes made to the original version. You should upload this as a separate file labeled 'Revised Manuscript with Track Changes'.
An unmarked version of your revised paper without tracked changes. You should upload this as a separate file labeled 'Manuscript'.

Response: All the 3 documents will be prepared and uploaded
If you would like to make changes to your financial disclosure, please include your updated statement in your cover letter. Guidelines for resubmitting your figure files are available below the reviewer comments at the end of this letter.

If applicable, we recommend that you deposit your laboratory protocols in [protocols.io](http://protocols.io) to enhance the reproducibility of your results. [Protocols.io](http://Protocols.io) assigns your protocol its own identifier (DOI) so that it can be cited independently in the future. For instructions see: <https://journals.plos.org/plosone/s/submission-guidelines#loc-laboratory-protocols>. Additionally, PLOS ONE offers an option for publishing peer-reviewed Lab Protocol articles, which describe protocols hosted on [protocols.io](http://protocols.io). Read more information on sharing protocols at <https://plos.org/protocols?utm_medium=editorial-email&utm_source=authorletters&utm_campaign=protocols>.

We look forward to receiving your revised manuscript.

Kind regards,

Bereket Yakob, Ph.D.
Academic Editor
PLOS ONE

Journal Requirements:

When submitting your revision, we need you to address these additional requirements.

======================================================================================================================================

1. Please ensure that your manuscript meets PLOS ONE's style requirements, including those for file naming. The PLOS ONE style templates can be found at
   <https://journals.plos.org/plosone/s/file?id=wjVg/PLOSOne_formatting_sample_main_body.pdf> and 
   <https://journals.plos.org/plosone/s/file?id=ba62/PLOSOne_formatting_sample_title_authors_affiliations.pdf>
   Response: The manuscript is made based on the PLOS ONE's style requirements
   2. Thank you for stating the following financial disclosure: 

   No specific funding available for this work.

   At this time, please address the following queries:

   a) Please clarify the sources of funding (financial or material support) for your study. List the grants or organizations that supported your study, including funding received from your institution. 
   b) State what role the funders took in the study. If the funders had no role in your study, please state: “The funders had no role in study design, data collection and analysis, decision to publish, or preparation of the manuscript.”
   c) If any authors received a salary from any of your funders, please state which authors and which funders.
   d) If you did not receive any funding for this study, please state: “The authors received no specific funding for this work.”

Response: The authors received no specific funding for this work. Authors voluntarily participated during data collection and any stage of the analysis report writing and manuscript preparation. This statement is also explained in the manuscript in the funding section.

Please include your amended statements within your cover letter; we will change the online submission form on your behalf. https://orcid.org/0000-0002-4857-5802

3. PLOS requires an ORCID iD for the corresponding author in Editorial Manager on papers submitted after December 6th, [2016](tel:2016). Please ensure that you have an ORCID iD and that it is validated in Editorial Manager. To do this, go to ‘Update my Information’ (in the upper left-hand corner of the main menu), and click on the Fetch/Validate link next to the ORCID field. This will take you to the ORCID site and allow you to create a new iD or authenticate a pre-existing iD in Editorial Manager. Please see the following video for instructions on linking an ORCID iD to your Editorial Manager account: <https://www.youtube.com/watch?v=_xcclfuvtxQ>

Response: Thank you greatly. The ORCID that I was using previously is 0000-0003-2882-5998. The submission system of the editorial manager is updated with this number.

4. Please include your tables as part of your main manuscript and remove the individual files. Please note that supplementary tables (should remain/ be uploaded) as separate "supporting information" files
Response: The table is included in the manuscript main body in **socio demographic sub section**

[Note: HTML markup is below. Please do not edit.]

Reviewers' comments:

Reviewer's Responses to Questions

Comments to the Author

1. Is the manuscript technically sound, and do the data support the conclusions?

The manuscript must describe a technically sound piece of scientific research with data that supports the conclusions. Experiments must have been conducted rigorously, with appropriate controls, replication, and sample sizes. The conclusions must be drawn appropriately based on the data presented.

Reviewer #1: Yes

Reviewer #2: Yes

2. Has the statistical analysis been performed appropriately and rigorously?

Reviewer #1: No

Reviewer #2: Yes

3. Have the authors made all data underlying the findings in their manuscript fully available?

The PLOS Data policy requires authors to make all data underlying the findings described in their manuscript fully available without restriction, with rare exception (please refer to the Data Availability Statement in the manuscript PDF file). The data should be provided as part of the manuscript or its supporting information, or deposited to a public repository. For example, in addition to summary statistics, the data points behind means, medians and variance measures should be available. If there are restrictions on publicly sharing data—e.g. participant privacy or use of data from a third party—those must be specified.

Reviewer #1: Yes

Reviewer #2: Yes

4. Is the manuscript presented in an intelligible(comprehensive) fashion and written in standard English?

PLOS ONE does not copyedit accepted manuscripts, so the language in submitted articles must be clear, correct, and unambiguous. Any typographical or grammatical errors should be corrected at revision, so please note any specific errors here.

Reviewer #1: No

Reviewer #2: Yes

5. Review Comments to the Author

Please use the space provided to explain your answers to the questions above. You may also include additional comments for the author, including concerns about dual publication, research ethics, or publication ethics. (Please upload your review as an attachment if it exceeds 20,[000](tel:000) characters)

Reviewer #1: This is an interesting paper that would add knowledge to the HRH regulation and healthcare safety.

Please find comments for further development.

Title: the study doesn’t include national-level managers and accreditors. As such, I would add “subnational level”
Authorship: 26 authors are much for such type of articles. Also, check authors’ contributions according to the International Committee of Medical Journal Editors (ICMJE) recommendations.

Response: Thank you for the comments. Because we did not have budget to do the study, we decided the study to do in group volunterly. Thus, all the authors participated in the key roles of the study such as drafting, tool preparation, data collection, data cleaning, and quality assurance, data and as it is described in the authors’ contribution section.

Abstract: lines [46-47](tel:46-47) and [54-55](tel:54-55) specify the results and key home-take recommendations
Response: Yes lines [46-47](tel:46-47) and [54-55](tel:54-55) specify the results and key home-take recommendations. Kindly we are not clear what to correct with this comment.

Background
1. The problem statement and rationale of the study is not clear.

Response.Thank you for this wonderful comment.we re-arranged it as per the next comment.Therefore the problem statement is at the first paragraph and the rationale is at the end of the background section
2. Paragraphs 1, 3, and 5 are only single sentences that do not fulfil the principle of paragraphing. In addition, please limit your citations to low-and-middle-income countries. As such, you may drop the 3rd paragraph.

Response:.We re-arraged the single sentences to the similar contents that can form a meaningful paragraph . The 3rd paragraph is dropped.And the flow started from global problem followed by LMICs and finally Ethiopia.The problem in developed countries is removed.
3. I would reorganize the Introduction section for smooth flow. Describe the problem statement in the first paragraph; the second paragraph could be about national HRH regulation standards and strategies; the third paragraph would focus on the HRH regulation shortfalls (including HRH shortages); and the fourth paragraph could be knowledge gaps and rationale of this study.

Response: Thank you again. We put all as per your suggestion. The first paragraph focus about problem statement; the second paragraph focus on national HRH regulation standards and strategies; the third paragraph focus on the HRH regulation shortfalls and the fourth paragraph on rationale of this study.
Methods
1) Setting: I would describe the profile of major health cadres of the country for external readers.

Response: Described in the manuscript, in Study design and setting section.
2) Any software used for data analysis?

Response: No software was used for analysis. It is done by manual coding, categorizing and analysis.
3) Comparative analysis to compare variations as a multiple case study approach amongst HR managers and accreditors would be helpful. For instance, awareness of licensing practices could not be a theme among accreditors

Response:The participants in this study were human resource managers and health professional regulators,not accriditators. Kindly, if you can suggest on the name of the theme, we can come to that.
4) Limitations: What would be the limitations of not including non-managers/practitioners’ perspectives in this study? I would reflect on this in the Discussion section

Response: We included an idea in the manuscript stating that ‘Not including non-managers/practitioners’/ perspectives in this study might lead to a limited understanding of the licensing practice at hand and lack of diversity in the data collected and analyzed’
Results
1. Lines [179-189](tel:179-189) are about the implementation fidelity (reliability) of the licensure or implementation challenges. I don’t think it is related to awareness of licensure

Response: Thank you for your detailed view. We removed from awareness and put them at the beginning of technical challenges
2. Lines [206-217](tel:206-217), the way presented here is about challenges rather than enforcement mechanisms.

Response: Narrative and quotation from 206-211 are moved to organizational challenges. Quotation from 213-217 moved to technical challenges, but to protect redundancy, we removed the narrative.

3. Lines [237-248](tel:237-248) better fits “Systems for assuring the quality of licensing practices”

Response: We transferred them as per your valuable comment. Thank you!
4. Sub-themes of the “Challenges of licensing practice” are broad and vague, and some of the paragraphs are not directly related. For instance, lines [276-281](tel:276-281) are not related to organizational problems. Similarly, lines [301-307](tel:301-307) are not also technical challenges. Rather it is a fraud, an individual behavior. I would recategorize the challenges as 1) fraudulent academic credentials, 2) poor coordination and network between the human resource department and regulatory body, 3) shortage of resources (HR, finance, equipment, and supplies), 4) lack of training or SBCC about licensing, and 5) weak support and governance system
Response: Rearrangment has been done. Sub themes are modified to 1.fraudulent academic credentials, 2.shortage of resources (HR, finance, equipment, and supplies) and 3. weak follow up and coordination system.

Discissions
1. Often in scientific writing, the first paragraph is dedicated to a summary of key findings and then subsequent paragraphs would compare each key finding with previous literature and discuss implications.
2. Lines [359-364](tel:359-364) and [377-380](tel:377-380) looks like repetitions of results and good to drop

Response: We dropped them
References: Check the references again reference # 22, 23, and 31 are not complete

Response: References ammended
Language: it has multiple grammatical errors that should be fixed; would benefit from experienced editors

Reviewer #2: Workforce licensing practice in Ethiopia: A qualitative study
Date 6 April [2023](tel:2023)
Dr. Mengistu Meskele
General comment
This is original research and a very important area of research in the health system quality in Ethiopia. It will befit for policy and program aspects. It is well written and triangulated with mixed methods research, is a strength. However, I have the following few comments.

Response: Dear reviewer, thank you for your positive views in the contribution of the paper.
1. Abstract: The word count should not exceed [300](tel:300)/[350](tel:350), as your abstract word count was [380](tel:380). I think that exceeds the journal requirement. Also, include keywords at the end of the abstract.

Response: We revised the abstract without changing its meaning and message. The word count now is 289.Key words are also included.
2. In the abstract section, the author has to include the sample size and techniques for qualitative study and the brief scientific rigours (credibility, dependability and transferability ). How they come across to maintain the quality of the study has to be included.

**Response**: Kindly,sample size(interviews with a total of 32 purposively selected health system managers). and other techniques were described. Member check was done to increase the (credibility, dependability and transferability ) of the study. we are happy to include if we are commented specifically to add more.

3. In line 48 the heading has to only ‘’conclusion’’ and word recommendation should be removed.

Response: Thank you! The heading recommendation is removed as per the comment.
4. Line 51” a reliable system to continuously assure the quality of licensing practice ‘’ Kindly put the reliable system that your research found. Otherwise, it is still broad and does not explicitly indicate the solution to policymakers. What is a reliable system? In line 55, encouraging interventions are recommended to respond to the above-identified challenges’’ has to be removed as it does not indicate the other intervention proposed.
Response: The reliable system is modified to reliable digital system since the digital system can have a database that can handle data consistently to show where the defect is. As per the comment, line 55 is removed. One another comment is added that says ’’Further awareness on licensing practice should be created especially for lower level employers’’.

Background
Line: 64 ‘’US’’ I think it is the United States, and all the rest abbreviation has to be written in full at the first encounter and the beginning of the sentences. Consider this comment for all the rest abbreviations. Example Line 80 LMICs

Response: All corrected and applied accordingly for all contents of the manuscript.

Line 55’’Other

Response: Kindly,it is done (removed) at 4^th^ comment

Line [140 -144](tel:140%20-144) has to be moved to the result section as how many themes you have identified.

Response: thank you! We did it accordingly.We have put the moved content next to socio demographic characteristics.

Line [145](tel:145): Phenomenological analysis framework??? What is that? Have you used Framework analysis / Collazi phenomenological? Not clear?

Response: Sorry, we corrected it by ‘We used thematic content analysis to analyse the result’.
Line [115](tel:115): Under quality control, no scientific rigors are included and maintained in this qualitative research (Trustworthiness, credibility, dependability, flexibility, transferability). Also, no reference was mentioned from where the author got the data collection tool were developed.

Response: Member check was done to assure the credibility of the study. Previously, it was explained in the data analysis section and now we shift it to the data collection and quality assurance section after your valuable comments. The data collection tool was developed by the authors based on the objectives of the study from the available different literatures and we did not need to cite a reference.

Kindly fill the: Consolidated criteria for reporting qualitative research (COREQ) for this research???
Response: COREQ filled accordingly
Results
Kindly describe themes after the sociodemographic sections

Response: thank you! Done accordingly.
Discussion: Revisited
Some paragraphs are not supported with body of [articles.For](http://articles.For) example [381-384](tel:381-384). Kindly support your discussion points with references from other research.

Response: Now we supported it by other similar finding in study Lao People's Democratic Republic.
References
Are not complete, and editorial and typographical errors. URL and Accessed date were not included for the grey literature. For example, Reference numbers: 19,21,20,22,23,27,31 etc, are not correct.

Response:Thank you ! Updated

6. PLOS authors have the option to publish the peer review history of their article (what does this mean?). If published, this will include your full peer review and any attached files.
Response: Yes

If you choose “no”, your identity will remain anonymous but your review may still be made public.


Do you want your identity to be public for this peer review? For information about this choice, including consent withdrawal, please see our Privacy Policy.

Reviewer #1: Yes: Gizachew Tadele Tiruneh

Reviewer #2: Yes: Mengistu Meskele

 


[NOTE: If reviewer comments were submitted as an attachment file, they will be attached to this email and accessible via the submission site. Please log into your account, locate the manuscript record, and check for the action link "View Attachments". If this link does not appear, there are no attachment files.]

While revising your submission, please upload your figure files to the Preflight Analysis and Conversion Engine (PACE) digital diagnostic tool, <https://pacev2.apexcovantage.com>/. PACE helps ensure that figures meet PLOS requirements. To use PACE, you must first register as a user. Registration is free. Then, login and navigate to the UPLOAD tab, where you will find detailed instructions on how to use the tool. If you encounter any issues or have any questions when using PACE, please email PLOS at [figures@plos.org](mailto:figures@plos.org). Please note that Supporting Information files do not need this step.

 

 


In compliance with data protection regulations, you may request that we remove your personal registration details at any time. (Remove my information/details). Please contact the publication office if you have any questions.
