# Supplementary material for: Health care managers’ perspectives on workforce licensing practice in Ethiopia: A qualitative study
Source: PLoS One. 2024 Apr 18;19(4):e0302122. doi: 10.1371/journal.pone.0302122 (PMC11025822; doi:10.1371/journal.pone.0302122)
Supplement: Attachment — Submitted filename: Review comments.docx [file pone.0302122.s004.docx]

General comments

- The manuscript requires thorough grammar and punctuation editing. There are many typos, and it will be difficult for the audience to follow the content. Language editing is highly recommended to ensure clarity and readability.
- List of authors: Confirm the contributions of every author. Follow PLOS One authorship guidelines. You can find it here <https://journals.plos.org/plosone/s/authorship#loc-authorship-requirements>

Abstract:

- Background: State the problem in the background. It only seems your study was about the importance of professional licensing bodies, not the barriers.
- Please improve the grammar and readability of the text in the abstract. For instance, 1) in the methods, “An interview guide was prepared in English, translated to” this must be changed into “translated into.” 2) results: “awareness on licensing practices” needs to be changed into “awareness of licensing practices.”
- Did you use any software to analyze the qualitative data, or did you do it manually?
- “Lack of awareness among managers was reported especially at lower-level employers.” Lack of awareness of what? Please specify it.
- “While regulators were clear on the requirements, employers placed an unwarranted emphasis on ensuring their employees met government licensing requirements.” This is unclear! Please state it in a simple language.
- “Lack of a quality assurance mechanism was reported.” By whom? Quality assurance of what?
- Reorganize and present the results to the four themes. Now, they are scattered all over.
- Embolden the participants' lived experiences by showcasing not only the challenges but also their positive experiences.
- Conclusions: “This study reported a sub-optimal health professionals’ licensing practice in Ethiopia.” You did not tell us what the optimal licensing practices would be or were that were unpracticed. But here, you are saying it was sub-optimal.
- Some of the recommendations were far-fetched and were not from the study results. For instance, the lack of digital systems and the lack of collaboration and communications between regulators and employers were not in the results.
- What is the distinction between “regulators” and “employers”? Who were the employers? Who were the regulators?

Background:

- General, seek editing services and improve language in the manuscript. Otherwise, it is difficult to follow it.
- Line 74: “The Ministry of Health and Education” – Is/was there any ministry called this way? These are separate entities and must be called by their formal names, such as the Ministry of Health and the Ministry of Education.
- Lines 91-92: “Therefore, this study aimed to explore the practice 92 and challenges of health professionals' licensing among Health care managers in Ethiopia.” The practice was not given due attention in the manuscript. The focus was simply on the challenges. You need to revise the above sentence or ensure sufficient details about the practices are included in the manuscript.

Methods

- Insert references for notable methods borrowed from elsewhere.
- Edit language and address typos.
- Lines 112-114: What was the rationale for selecting 12 regional HR and 12 regional regulatory managers? Why only 4 zonal and 4 district HR managers? What was the rationale for the sampling strategy, and what was the purpose of the purposive sampling? Discuss this further.
- What were the roles of zones and districts regarding licensing and regulation? Discuss this further.
- What was the reason for excluding the national licensing and regulatory bodies from the study? Do you need to revise the study topic accordingly?
- Lines 114-116: “Authors had access to information that could identify individual participants can be identified during or after data collection by their labelling as (regional regulatory manager 1 etc.).” The sentence is unclear! How did you ensure the ethical issues about this? Didn’t this endanger the confidentiality and privacy of the participants? Why did you not anonymize the data before sharing it with all authors? Discuss and correct it if there is a reasonable ground for you to share identifiable data with all authors.
- Line 119: Who prepared the interview guide? How did you ensure its content validity?
- Line 153: “We used thematic content analysis to analyse the result.” Discuss the method in brief and insert references. This must come early when you talk about the methods.
- Did you use any software to assist with handling and analyzing the qualitative data?
- Line 145-146: “Coding was done by 146 eight of the investigators (authors). Codes were given for emerging ideas.” Too many people coded the data! How did you ensure variations in coding and analyzing the data? How did it impact interpretation?
- Line 154: It seems something is misplaced or missing.
- Lines 157-158: “In this study, health care managers were represented by regulatory managers 158 and human resource managers in Ethiopia.” The sentence is unclear!

Results

- Lines 171-172: “The highest proportions 172 (75 %) of participants were from the regional level.” Rephrase the sentence.
- Lines 195-196: Were the themes there, or did they emerge during the analysis and interpretation? How distinct are the themes from each other? For instance, what was the difference between “enforcement of licensing practices” and “Systems for assuring the quality of licensing practices”? Were they not more or less similar?
- Lines 212-244: Nothing here seemed to be “enforcement of the licensing practice.” It was simply procedures the licensing bodies followed to grant licensure to healthcare workers. Define what you meant by “enforcement” and discuss its attributes. Show appropriate exhibits as evidence for it.
- Lines 245-280: What is written here is more about the availability and practices of regulatory mechanisms. However, the data were not synthesized to a higher-level theme. Further analysis, synthesis, and interpretation will be helpful.
- Line 281+: Besides what is said in this section, many challenges are mentioned in lines 212-280. For instance, lack of awareness and orientation for staff on licensure procedures and regulatory mechanisms, unavailability of digital systems, poor planning and execution, etc.” Further synthesis, theme development, and interpretation need to be done.
- Lines 320-324: How has this become a health system problem?
