# Supplementary material for: Health care managers’ perspectives on workforce licensing practice in Ethiopia: A qualitative study
Source: PLoS One. 2024 Apr 18;19(4):e0302122. doi: 10.1371/journal.pone.0302122 (PMC11025822; doi:10.1371/journal.pone.0302122)
Supplement: Attachment — Submitted filename: Response to reviewers- line by line- 4.docx [file pone.0302122.s005.docx]

General comments

- The manuscript requires thorough grammar and punctuation editing. There are many typos, and it will be difficult for the audience to follow the content. Language editing is highly recommended to ensure clarity and readability.
- Response: All parts of the manuscript is checked and corrected for grammar, punctuation and errors
- List of authors: Confirm the contributions of every author. Follow PLOS One authorship guidelines. You can find it here <https://journals.plos.org/plosone/s/authorship#loc-authorship-requirements>

Response: Checked

Abstract:

- Background: State the problem in the background. It only seems your study was about the importance of professional licensing bodies, not the barriers.

Response፡ Thank you! A statement describing the barriers for health workforce licensing practice. is included in the manuscript.

- Please improve the grammar and readability of the text in the abstract. For instance, 1) in the methods, “An interview guide was prepared in English, translated to” this must be changed into “translated into.” 2) results: “awareness on licensing practices” needs to be changed into “awareness of licensing practices.”

Thank you! Done as per the comments.

- Did you use any software to analyze the qualitative data, or did you do it manually?

Response: We don’t use software; rather we analyzed it manually and explained in the result section of the manuscript.

- “Lack of awareness among managers was reported especially at lower-level employers.” Lack of awareness of what? Please specify it.
- Response: awareness about health workforce licensing is explained in the manuscript.
- “While regulators were clear on the requirements, employers placed an unwarranted emphasis on ensuring their employees met government licensing requirements.” This is unclear! Please state it in a simple language.
- Response: Thank you, we made it a clear statement.
- “Lack of a quality assurance mechanism was reported.” By whom? Quality assurance of what?
- Response: Greatly thank you! We tried to make it clear by substituting this unclear statement by “health care managers mentioned that they did not know any monitoring tool to solve the issue of working without a license”.
- Reorganize and present the results to the four themes. Now, they are scattered all over.
- Response: Results: We think that we made the results in the order we explained the four major themes: awareness of licensing practices, enforcement of licensing practices, systems for assuring the quality of licensing practices, and challenges to licensing practices.
- The first statement is about awareness- Lack of awareness among managers about health workforce licensing was reported especially at lower level employers.
- The second statement is about enforcement of licensing practices- Regulators were clear on the requirements to issue a license for health workforce if they are competent the licensing exam while human resource managers do not give emphasis whether the employees have a license or not during employment. As a result of this, non-licensed health work forces were employed.
- The third statement is about systems for assuring the quality of licensing practices: Health care managers mentioned that they did not know any monitoring tool to solve the issue of working without a license.
- The last statement is about challenges to licensing practices: Lack of a quality assurance mechanism was reported. Fraudulent academic credentials, shortage of resources (human resource, finance, equipment, and supplies) and weak follow up and coordination system were identified as main practice challenges.
- Embolden the participants' lived experiences by showcasing not only the challenges but also their positive experiences.
- Response: Ok ! done
- Conclusions: “This study reported a sub-optimal health professionals’ licensing practice in Ethiopia.” You did not tell us what the optimal licensing practices would be or were that were unpracticed. But here, you are saying it was sub-optimal.
- Response: Thanks it is a visible comment! The optimal licensing practice is described
- Some of the recommendations were far-fetched and were not from the study results. For instance, the lack of digital systems and the lack of collaboration and communications between regulators and employers were not in the results.

Response:

- The lack of digital systems. The result section for this conclusion statement is derived from the the theme “Challenges of licensing practice “, subtheme “Shortage of resources (human resource, finance, equipment, and supplies) “, line 326. The lack of information communication materials and trained professional describes the digital system.
- The lack of collaboration and communications between regulators and employers. The result section for this conclusion statement is derived from

the theme “Challenges of licensing practice “, subtheme “Weak follow up and coordination system” line number 354.

- What is the distinction between “regulators” and “employers”? Who were the employers? Who were the regulators?

Response: Regulators are authorized personnels working in the regulatory offices (FMHACA) of Ethiopia. Employers are human resource managers in the health care system.

Background:

- General, seek editing services and improve language in the manuscript. Otherwise, it is difficult to follow it.

Response: Punctuation and grammar checked, edited and corrected.

- Line 74: “The Ministry of Health and Education” – Is/was there any ministry called this way? These are separate entities and must be called by their formal names, such as the Ministry of Health and the Ministry of Education.

Response: Done as per the comment

- Lines 91-92: “Therefore, this study aimed to explore the practice 92 and challenges of health professionals' licensing among Health care managers in Ethiopia.” The practice was not given due attention in the manuscript. The focus was simply on the challenges. You need to revise the above sentence or ensure sufficient details about the practices are included in the manuscript.
- Response: Thank you! Practice is substituted by perspectives. This is because awareness on licensing practices, enforcement of licensing practices, Systems for assuring the quality of licensing practices discuss about perspectives of health work force licensing.

Methods

- Insert references for notable methods borrowed from elsewhere.
- Response: reference for member checking is cited
- Edit language and address typos.
- Response: Ok! done
- Lines 112-114: What was the rationale for selecting 12 regional HR and 12 regional regulatory managers? Why only 4 zonal and 4 district HR managers? What was the rationale for the sampling strategy, and what was the purpose of the purposive sampling? Discuss this further.

Response: the below reasons are included in the manuscript

- The rationale for selecting 12 regional HR and 12 regional regulatory managers is because there are 12 regional states in Ethiopia during data collection period.
- Four zonal and four district HR managers were selected purposively based on the regional burden of licensing issue problem based on the national data.
- The rationale behind using purposive sampling in this study is to select a sample of health care managers who can most likely provide the perspective regarding the health work force licensing practice in Ethiopia needed to answer the research question. Purposive sampling is often used when the population of interest is small, and we used purposing sampling because we want to focus in-depth on relatively small samples. We used it because we want to access these key informant health workforces that shared us their experiences.
- What were the roles of zones and districts regarding licensing and regulation? Discuss this further.

Response:

Zones Health Departments-

- Receive direction, circular letter from the regional health Bureau and implement accordingly
- Issue a license for health workforce and execute the licensing and regulation system
- Employ health workforce for zonal and district level by Human resource department
- Inspect the licensing status of health work force and other regulatory requirements at zonal and district l level as per the standard
- Archiving licenses and other documents
- Reporting feedbacks to regional level.

District Health Offices

- Receive direction, circular letter from Zonal Health Department and implement accordingly
- Execute the licensing and regulation system at district and facility level
- Employ health workforce by Human resource department for district health office and health facilities
- Inspect the licensing status of health work force and other regulatory requirements at district and facility level as per the standard
- Archiving licenses and other documents
- Reporting feedbacks to zonal level.
- What was the reason for excluding the national licensing and regulatory bodies from the study? Do you need to revise the study topic accordingly?
- Response: The study aimed to dig-out ideas from regional and lower levels since improvements could be achieved if practices and challenges at the lower levels are understood.
- Lines 114-116: “Authors had access to information that could identify individual participants can be identified during or after data collection by their labeling as (regional regulatory manager 1 etc.).” The sentence is unclear! How did you ensure the ethical issues about this? Didn’t this endanger the confidentiality and privacy of the participants? Why did you not anonymize the data before sharing it with all authors? Discuss and correct it if there is a reasonable ground for you to share identifiable data with all authors.

Response: I appreciate your view & thank you! It is our explanation problem. In fact it is to identify the responses of regional, zonal and districts participants finally to send our result summary for member checks. Nobody knows who “regional regulatory manager 1” is. We anonymized the data by removing any identifying information to protect their data is not misused finally. To prevent unauthorized access or use, their data is stored securely.

-Correction is made in the manuscript.

- Line 119: Who prepared the interview guide? How did you ensure its content validity?
- Response: The interview guide was prepared by the authors in a team who are involved in the study. The guide was a comprehensive and covers all parts our objectives want to explore. It encouraged participants to share their experiences in their own words and in their own way without being forced by categories or classifications imposed by the interviewer.
- Correction is made in the manuscript.
- Line 153: “We used thematic content analysis to analyse the result.” Discuss the method in brief and insert references. This must come early when you talk about the methods.
- Response: Discussed and referenced. Thank you!
- Did you use any software to assist with handling and analyzing the qualitative data?
- Response: No, we did not use software for analysis. We did it manually.
- This idea is added in the manuscript
- Line 145-146: “Coding was done by 146 eight of the investigators (authors). Codes were given for emerging ideas.” Too many people coded the data! How did you ensure variations in coding and analyzing the data? How did it impact interpretation?
- Response: Thank you for seeing the detail. We ensured variation in coding and analysing the data by having multiple coders to identify discrepancies and maintain consistency. Inter-coder reliability is established by comparing results from multiple coders. Disagreements are identified and resolved to maintain consistency. A senior researcher (among the authors) reviewed the coding and analysis process to ensure it aligns with the research question and results are reliable and valid.

Correction is done in the manuscript.

- Line 154: It seems something is misplaced or missing.

Response, we are ready to correct any missing or misplaced

- Lines 157-158: “In this study, health care managers were represented by regulatory managers 158 and human resource managers in Ethiopia.” The sentence is unclear!

Response: This is operational definition .It means that the population named in the study by health care managers includes regulatory managers and human resource managers in Ethiopia.

Results

- Lines 171-172: “The highest proportions 172 (75 %) of participants were from the regional level.” Rephrase the sentence.

Response: Rephrased

- Lines 195-196: Were the themes there, or did they emerge during the analysis and interpretation? How distinct are the themes from each other? For instance, what was the difference between “enforcement of licensing practices” and “Systems for assuring the quality of licensing practices”? Were they not more or less similar?

Response:

The themes were first there , but their name is modified during review of the paper as per the recommendation of reviewers.

There is a difference between “enforcement of licensing practices” and “Systems for assuring the quality of licensing practices”. The first is about implementation of health work force licensing while the second is about monitoring, evaluation system and other quality assurance related issues.

- Lines 212-244: Nothing here seemed to be “enforcement of the licensing practice.” It was simply procedures the licensing bodies followed to grant licensure to healthcare workers. Define what you meant by “enforcement” and discuss its attributes. Show appropriate exhibits as evidence for it.
- Response: enforcement of the licensing practice means the implementation of licensing for health workforce. Perhaps if there is any other better naming we can amend it.
- Lines 245-280: What is written here is more about the availability and practices of regulatory mechanisms. However, the data were not synthesized to a higher-level theme. Further analysis, synthesis, and interpretation will be helpful.

Response : Ok!

- Line 281+: Besides what is said in this section, many challenges are mentioned in lines 212-280. For instance, lack of awareness and orientation for staff on licensure procedures and regulatory mechanisms, unavailability of digital systems, poor planning and execution, etc.” Further synthesis, theme development, and interpretation need to be done.
- Response: Thank you! Could you please give us a hint ?
- Lines 320-324: How has this become a health system problem?

Response: This paragraph tells us there is lack of money and lack of transportation to travel to regional levels to take a license. For this reason, if health professionals are not taking their license, they will continue to work with their expired license, forced to have forged license, that has a direct impact on the delivery of the health care service which is part of the health system.
