# Supplementary material for: Health care managers’ perspectives on workforce licensing practice in Ethiopia: A qualitative study
Source: PLoS One. 2024 Apr 18;19(4):e0302122. doi: 10.1371/journal.pone.0302122 (PMC11025822; doi:10.1371/journal.pone.0302122)
Supplement: Attachment — Submitted filename: Line by line response to 5th.docx [file pone.0302122.s006.docx]

PONE-D-22-33343R2

Health care managers’ perspectives on workforce licensing practice in Ethiopia: A qualitative study

PLOS ONE

Dear Dr. Teka,

Thank you for submitting your manuscript to PLOS ONE. After careful consideration, we feel that it has merit but does not fully meet PLOS ONE’s publication criteria as it currently stands. Therefore, we invite you to submit a revised version of the manuscript that addresses the points raised during the review process.

==============================
**ACADEMIC EDITOR:**

Lines 72 – 75: FMHACA was reformed to a different organization. Which organization or health agency was responsible for managing health workers' licensure when the study was done? Discuss how FMHACA (or another organization) and the MOH streamline the licensure practice. Based on your presentation, who was responsible for the task is unclear.

Response: Thank you, now we have reframed it in a clear way. The candidates, the types of exams and their level and the authority given to issue a license is stated in a clear way.

Line 88: Remove the unnecessary parenthesis and full stop.

Response:Thank you! Unnecessary parenthesis and full stop are removed

Lines 100-101: “This study was conducted in Ethiopia, a 101 country in Africa with an estimated population of 110 (15).” Something is missing here – Did you mean 110 million?

Response: Corrected. It is to say 110 million

Lines 107 – 111: Cite references.

Response: Reference cited

Line 105: Remove “o” at the end of the sentence.

Response: O is removed at line 115

Lines 118-120: Cite references

Response: Reference is cited

Lines 122 – 128: How many participants from regions, zones, and districts were included in the study? Mention the names of the regions that participated in the study or justify not mentioning it. If there were good reasons for hiding the details, use codes to represent the regions and other structures and present the data. At least provide summaries of the number of regions, zones, and woredas that participated and the number of people who participated from each region and structure. Align this with Table 1 (Line 204).

Response: Corrected accordingly

Line 128: “Number We anonym zed the data by removing any identifying…” The word anonymized was broken, and there were typos.

Response: Corrected accordingly

Lines 206 – 359: The results happen to be raw. A high-level conceptualization and synthesis might be useful.

Response: A slight modification in grammar,

==============================

Please submit your revised manuscript by Mar 22 2024 11:59PM. If you will need more time than this to complete your revisions, please reply to this message or contact the journal office at [plosone@plos.org](mailto:plosone@plos.org). When you're ready to submit your revision, log on to <https://www.editorialmanager.com/pone/> and select the 'Submissions Needing Revision' folder to locate your manuscript file.

Please include the following items when submitting your revised manuscript:

- A rebuttal letter that responds to each point raised by the academic editor and reviewer(s). You should upload this letter as a separate file labeled 'Response to Reviewers'.
- A marked-up copy of your manuscript that highlights changes made to the original version. You should upload this as a separate file labeled 'Revised Manuscript with Track Changes'.
- An unmarked version of your revised paper without tracked changes. You should upload this as a separate file labeled 'Manuscript'.

If you would like to make changes to your financial disclosure, please include your updated statement in your cover letter. Guidelines for resubmitting your figure files are available below the reviewer comments at the end of this letter.

If applicable, we recommend that you deposit your laboratory protocols in [protocols.io](http://protocols.io/) to enhance the reproducibility of your results. Protocols.io assigns your protocol its own identifier (DOI) so that it can be cited independently in the future. For instructions see: <https://journals.plos.org/plosone/s/submission-guidelines#loc-laboratory-protocols>. Additionally, PLOS ONE offers an option for publishing peer-reviewed Lab Protocol articles, which describe protocols hosted on [protocols.io](http://protocols.io/). Read more information on sharing protocols at <https://plos.org/protocols?utm_medium=editorial-email&utm_source=authorletters&utm_campaign=protocols>.

We look forward to receiving your revised manuscript.

Kind regards,

Bereket Yakob, Ph.D.
Academic Editor
PLOS ONE

Journal Requirements:

Please review your reference list to ensure that it is complete and correct. If you have cited papers that have been retracted, please include the rationale for doing so in the manuscript text, or remove these references and replace them with relevant current references. Any changes to the reference list should be mentioned in the rebuttal letter that accompanies your revised manuscript. If you need to cite a retracted article, indicate the article’s retracted status in the References list and also include a citation and full reference for the retraction notice.

Additional Editor Comments:

Check typos and address them. There are errors in almost all sections. The manuscript may benefit from proofreading and editing services.


[NOTE: If reviewer comments were submitted as an attachment file, they will be attached to this email and accessible via the submission site. Please log into your account, locate the manuscript record, and check for the action link "View Attachments". If this link does not appear, there are no attachment files.]

While revising your submission, please upload your figure files to the Preflight Analysis and Conversion Engine (PACE) digital diagnostic tool, <https://pacev2.apexcovantage.com/>. PACE helps ensure that figures meet PLOS requirements. To use PACE, you must first register as a user. Registration is free. Then, login and navigate to the UPLOAD tab, where you will find detailed instructions on how to use the tool. If you encounter any issues or have any questions when using PACE, please email PLOS at [figures@plos.org](mailto:figures@plos.org). Please note that Supporting Information files do not need this step.

*In compliance with data protection regulations, you may request that we remove your personal registration details at any time.* [*(Remove my information/details)*](https://www.editorialmanager.com/pone/login.asp?a=r)*. Please contact the publication office if you have any questions.*
